# On Hæmatocele Retro-Uterina

**Published:** 1864-03

**Authors:** Rud. Ferber

**Affiliations:** Hamburg


ART. I.—*On Hæmatocele retro-uterina, by Dr. Rud. Ferber in Hamburg; translated from the German by* H. LASSING, M. D., *New York.*

For the Buffalo Medical and Surgical Jeurnal.

Regarding this peculiar disease there have been latterly many investigations, particularly in France, the combined results of which appear to be of interest. For our present treatise we have taken for a basis the detailed treatise which *A. Voisin* first published as a thesis and afterwards as a monograph. (De l'hèmatocéle rètro-utèrine et des èpanchements sanguins non-enkystés de la cavité peritonèale du petit bassin, considérès comme accidents de la menstruation, par Aug. v. Paris, 1860, v. Masson, 8, viii 368 pp.) The views of several other authors upon the same subject are introduced in their proper places. *Voisin* begins with a historical introduction, which he divides into two periods, from the earlier times to *Nelaton*, (1850,) and from him, (who first gave an accurate definition of retro-uterine hæmatocele) down to the latest observations. Although there are exceptional cases, in which hæmatocele is found anterior to and lateral to the uterus, *Voisin* retains the original name of *Nelaton.* He describes a retro-uterine hæmatocele as an encysted sanguinous effusion into the peritoneal cavity of the small basin between the uterus and the rectum, (that is, the plica douglasii.) Hæmatocele according to his view is always consequent upon a disturbance of the menstrual function, and therefore always involves one of the organs which take part in the production of this secretion, namely the uterus, fallopean tubes or ovaries. All hæmorrhages arising from any blood vessel not belonging to this region are to be excluded, Effusions between the lig-lata are not to be included, as they may arise from other causes, particularly exertions of any kind. *Voisin* calls these thrombies. The vessels from which in this case the hæmorrhage arises, according to the anatomical proofs of *Voisin* are in no way connected with those which supply the above named organs. Also of the non-encysted sanguinous effusions into the peritoneal cavity of the small basin he has only considered those which are connected with the catameniæ.

A narrower boundary line is drawn by nearly all the other authors. Dr. *Albert Puech* (Journ. de Brux, xxxi, pp. 44, sq. Juillet—Nov. 1860,) understands by the term hæmatocele, which he divides according to its location as retro, pro, and latero-uterine, a tumor internal or external to the peritoneum arising from an effusion of blood in consequence of a lesion of one or more of the organs appertaining to the uterus. He claims that it is developed as well during as after the menstrual period, and even possibly during pregnancy. *Trousseau* (L'Union mèd 153–155, 1861,) also accepts the theory of an intra and extra peritoneal hæmatocele, and designates as main groups, an "hématocéle ovarienne," and "hèmatocèle tubaire or cataméniale." Dr. *T. Gallard* (Arch. génèr 5 Sér. xvi, p. 385, sq. Oct., Nov., Déc. 1860,) applies to those hæmorrhages which result from a functional disturbance of the sexual organs the term of "hématocèle spontanée;" and a sanguinous discharge consequent upon trauma or a mechanical hindrance he calls a "hèmatocéle chirurgicale." He denies the direct influence of menstruation. Also the bloody cysts he counts as a frequent first stage of the hæmorrhage spoken of among hæmatoceles. In his view the effusions can take place within as well as without the peritoneum.

Of the same view is Prof. *C. Braun* of Vienna, (Wiener med. Wochenschrift xi, 28, 29, 30, 34, 35, 1861.) He describes that which he calls "hæmatocele extra uterina," as an extravasation of blood in the surroundings of the uterus, an enclosure of this in a cyst and a consequent formation of a larger or smaller tumor which crowds itself down into the posterior, or lateral and sometimes also in the anterior, laquear and under certain conditions appears.

According to Dr. *Alfred Hegar* at Darmstadt, finally, (Mon. Schrift f. Geburtsk. xvii, p. 418, Juni, 1861,) we have now reached so far that we call all sanguinous effusions in the female pelvis by the name of hæmatocele peri-uterina. In extra peritoneal effusions the peritoneum is either only lifted up or its folds separated, or it may even be torn loose from the uterus. By a rupture of the peritoneum, according to Puech, the external hæmorrhagic effusion can become intra-peritoneal. Extra-peritoneal hæmatocele according to Hegar has as a boundary below, the pelvic fasciae, with this is connected the fascia transv, abd. and iliaca, for this reason sanguinous effusions in the fossa iliaca below the peritoneum can descend to the fascia pelv. The term thrombus, is now rather more confined to sanguinous effusions below the fascia pelv., which are mostly situated around the vaginal canal.

*Voisin* distinguishes two forms of retro-uterine hæmatocele; the simple, where the effused blood is entirely re-absorbed. and the complicated, where the blood is decomposed and mostly seeks an outward passage. Unencysted hæmorrhages, etc., divides into active and passive; both of which seldom occur. After a review of the different theories regarding the origin of retro-uterine hæmatocele, of which we will speak hereafter.—Voisin turns to aetiology. As pre-disposing causes he mentions in particular the period between the 21st and 40th year of age, the time of the greatest activity of the generative system in the female; the composition of the blood, particularly the increase and decrease of the quantity of fibrin; menstruation, in 25 cases, this had commenced between the 14th and 16th years in the majority, of 13 women 9 menstruated regularly, 4 irregularly, in 17 cases 9 were always very profuse, 4 normal, in 4 clots were passed, in 10 was accompanied by pain, the duration varied considerably. Again, obstinate constipation, by the pressure of the bowels filled with hardened fæcces can produce the formation of varicose veins in the lig. lat. The formation of hæmatocele is also favored, as already mentioned, by the several diseases of the circulation, particularly the acute exanthemaes. It was observed in purpura, icterus gravis, scarlatina, morbillaes and variola. *Barlow*, *Simpson*, *M. Helie*, *Laboulbene*, and others, found the hæmorrhages confined to the tubes without any effusion into the peritoneum. *Scanzoni* and *Barlow*, upon the other hand, observed the actual supervention of a bloody tumor in the peritoneal sac. *Trousseau* calls this species cachectic hæmatocele. As motor causes, *Voisin* observed, that in 10 patients, in whose cases the first symptoms coincided with menstruation, 7 had had coitus during or one day after menstruation. As a cause of nonencysted sanguinous effusions, a rough exercise of the sexual act is particularly enumerated. According to *Gallard*, coitus, even without any excess, has at least as great if not a greater influence upon the origin of the diseases as the menses, for if these were the principal aetiological causes, the disturbance produced must necessarily be observed at the first appearance of menstruation, that is between the 15th and 18th year, but not in the 30th year. According to *Gallard* corporeal exertion of every nature will readily serve as a cause, particularly the rupture of a vessel; also during menstruation it may be caused by an irregular contraction of the fallopian tubes whereby the ova and blood therein contained is expelled into the peritoneal sac. Also mental emotions may cause this. *Hegar* mentions a case of a girl 18 years of age, where a severe cold was the cause. *Puech* gives us a similar case, but hæmatocele appeared much more rapidly in this case.

As the most frequent cause, however, we may nevertheless consider the sexual excitement during coitus. The erection of the clitoris is always accompanied by a considerable turgescence of the veins of the clitoris, uterus and ovariae, by which as well during as after menstruation the disposition to a rupture of a vessel is considerably increased.

Of many different kinds are the theories relating to the *origin* of the sanguinous tumor in the neighborhood of the uterus. The majority of the observers acknowledge as a principal cause an organic disturbance of the ovaries or a functional disturbance of the tubes. Many admit exclusively the one or the other of these origins. For instance, *Nelaton*, *Denonvilliers*, *Huguier Lenoir* and *Laugier* look at the ovarium as the source of the disease, and this at the time of ovulation, without any primary disease of the organ and outside of this period with preceding disease. Against this, *Trousseau*, in page 58, asserted that the mucous membrane of the fallopian tubes alone is to be considered the source. Now he has changed his views, and with *Puech*, *Voisin*, *Bernutz* and *Gallard*, admits a multiple aetiology of hæmatocele. Nevertheless, each one of these investigators tries to put one or the other of these theories into the front rank, and discussion has therefore not yet ended. *Voisin*, who has subjected all the different views to a stringent review in his work, claims three different causes for the condition known by him as hæmatocele retro-uterina : a congestion of the ovarian follicles and consequent hæmorrhage occurring during menstruation, the re-flow of the blood from the uterus into the tubes and peritoneal sac, and a hæmorrhage of the tubes. Hæmatocele, therefore, is only the result of such sanguinous effusions which arise from the mucous membrane of the uterus and tubes, and from the membrana propria of the Graffian follicles, a co-existent menstruation and the accompanying increased afflux of blood are always presumed to be present. In the normal state, the ovarian follicles are capable of effusing a certain quantity of blood at the moment of the expulsion of the ovum. An inevitable condition for the origin of an hæmatocele, is a previous disease of the organ. If this consists in the enlargement of one or more follicles, the adducting vessels also become hypertrophied and produce a more active circulation and a disposition to hæmorrhage. Analogous to hæmorrhages from other mucous membranes, a hæmorrhage may also arise from the mucous membranes of the tubes; a pre-disposition to this we find in the physiological sanguinous evacuation during menstruation. But as only two cases produced an encysted sanguinous extravasation in the peritoneum, it is proven that the hæmorrhages of the tubes cannot be considered as the only, nor even as the most frequent cause (*Puech*). Finally, under certain circumstances, the blood effused into the uterine cavity may re-flow into the tubes; to do this, there must be an obstruction to the expulsion of the blood through the canal. The only cause which appears possible to *Voisin* for hæmorrhages during menstruation, is flexion of the uterus. Of this kind, however, there has as yet been but one reliable case observed by *Trousseau*. All other authors nearly, include herewith all those cases in which a hereditary or accidental atresia of the uterus or vagina are present, or where an occlusion from coagulum in consequence of excessive menstruation or metrorrhagia supervened. *Trousseau*, in conjunction with *Velpeau*, observed a case of this kind.

We ought, also, to refer here to the observations of *Marchant* and *Mussi*, where there was imperforation of the os; also the case of *Decis*, where there was a uterus and double vagina without external orifice.

*Trousseau* adopts as principal varieties of hæmatocele the "accidental" ovarian, hæmorrhage, with primary disease of the organ, and the "catamenial" hæmorrhage of the tubes, without change in these. His reasoning is briefly as following: The diseased ovarium, or a therein contained blood cyst, requires an opportune cause in order to burst, and to effuse blood into the peritoneal sac. As causes, we find in addition to all sorts of lesions also, but not exclusivly, menstruation. This form is the more seldom. The principal cause of hæmorrhage of the tubes, on the contrary, is menstruation, which, according to the experiments of Rouget, necessitates a considerable hyperaemia of the mucous membrane of the ducts. But, as hæmorrhages from mucous membranes require for their origin only a congestion (or even only a change in the blood,) and not a disease of the elements composing the membrane, the tubes, in consequence of the frequent motor causes, (monthly afflux of blood,) are the most usual sources of hæmatocele. *Puech* also considers hæmorrhage of the tubes in the first rank. The view of *Tardieu*, who considered the peritoneal vessels as sources of the hæmorrhages, is admitted by *Trousseau* in carcinomatous or tuberculous peritonitis.

It only remains for us to refer to the theory of *Gallard* (originally that of *Viques*,) which is: the ovum at each menstruation differs in nothing from the condition in which it is found at beginning gravidity. The mechanism by which an hæmatocele peri-uterina arises, differs therefore in nothing from that, under the influence of which an extra uterine conception is formed. An hæmatocele peri-uterina always appears then where a graviditas extra-uterina may show itself. The separation and descent of an impregnated ovulum pre-disposes more to an hæmatocele than the same process with an unimpregnated ovulum. *Gallard*, by this hypothesis, claims to have explained all those which he calls spontaneous hæmatoceles, and lays the highly seldom occurrence of oval residues, either to the immense difficulties of discovering such, or to the careless researches of the observers, Among the chirurgical hæmatoceles, he includes those which arise from trauma ulceration and rupture of the coats of a vessel, or re-flow of the blood. *Voisin* proves this theory to be untenable, yet it is possible that an apoplexy of an ovum deposite intra-uterines may produce a sanguinous tumor, to which fact *Braun* also calls attention.

The *symptoms* of *hæmatocele retro-uterina*, independent of the entirely unobserved passive smaller extravasations, the residues of which form a much more frequent and unexpected subject of observation at autopsies, in the main, according to Voisin, a peritonitis arising from perforation with hæmorrhagic exudations. First we find an intense pain which, with the least pressure or the slightest movement, is much increased; this is located in the pelvic cavity, and is said to resemble labor pains. During the progress of the disease, the pain is increased at each menstruation. At first even there is formed a tumor in the pelvic cavity, which rises more or less towards the umbilicus, and is generally more voluminous in the right hypogastric region than in the left, and may extend itself to the iliac fossa. The sound upon percussion over the same is dull. During its first stage, the tumor is soft and fluctuating, later, of variable firmness, and, at times, becomes again fluctuating. By the touch we can plainly feel the tumor, the os is forced forward, the rectum flattened backward. The walls of the vagina are extended, the lower part of the same is generally occupied by the tumor, which, to the extent of eight inches, advances towards the orificium vulvæ. Micturition and defæcation are performed with difficulty.

The tumor, by pressure upon the pelvic nerves and vessels, may produce a corresponding pain and œdema of the extremities. The general symptoms are those of a peritonitis combined with those of an internal hæmorrhage. Clinically, according to *Braun* and the other authors, there is no deviation between the symptoms of an intra and extra-peritoneal hæmatocele. Very different and aggravated disturbances may be produced by sanguinous tumors in the course of a gestation or even a parturition. *Braun* bases the following conclusions upon several observations: Hæmatocele may produce a dangerous obstacle to parturition and a dimunition of the diameters; hæmatocele retrouterina may occur with accompanying prolapsus of the vaginal membrane; the sanguinous tumors may (during parturition) be immovable, if they expand in the retro-peritoneal redoublications of the uterus or of the vagina, or as intra peritoneal tumors have grown fast to these or even may remain movable if they are seated in the broad ligaments of the womb, the ovaries or the fallopian tubes.

The course of non-encysted sanguinous effusion is very acute, death mostly supervenes in about twelve hours. On the contrary, the course of an hæmatocele is very different. Sometimes it appears with great violence, and at other times we have only the appearance of a sub-acute morbid action, and sometimes it passes entirely unobserved. Mostly we find the tumor at the commencement has attained its full growth in a few cases. A growth of the same was observed during menstruation. *Gallard*, on the contrary, has observed a considerable diminution at this period. Generally, it soon acquires a hard consistency, and the fluctuation gradually disappears. The effused blood separates into serum and fibrin; both are gradually absorbed, or the latter decomposes, commingles with an exudation supplied by the peritoneum, and thus forms a mass resembling current-jelly, which finally forms its way out. In duration, it differs very much. *Generally*, according to *Puech*, extra peritoneal effusions run a more rapid course than those intra peritoneal; also the smaller are quicker than the larger. Fatal results, compared to recovery, seldom occur.

If the effusion is left to nature it may have the following results: (*a*) re-absorption; (*b*) discharge through the rectum, vagina or into the peritoneal sac; (*c*) dispersion. *Dr. F. Bockelmann*, of Bremen (Wién. Med. Halle I 20, 1061) gives us a very accurate description of the local changes just pointed out as resulting from a sanguinous extravasation. The thickened mass is often very slowly re-absorbed, and therefore we can feel the tumor for a long time at least from the vagina. The decomposition and formation of pus is generally a very bad complication; it may undoubtedly materially abreviate the duration of the disease; but on the other hand, give origin to very imminent dangers. According to *Puech* discharges took place in 46 cases as follows: 4 through the vagina, in which the results were always favorable, the extravasation disappeared after 24 to 48 hours, but also lasted in one, 8 to 14 days; in two cases discharge took place at two different periods, separated by a considerable interval; ten times through the rectum, less favorable, including three deaths, and lasting in some cases over a month; five times into the peritoneal cavity, all fatal. All these cases occurred without the interference of art, and of the others 22 recovered by re-absorption.

Regarding the *diagnostic points*, which of course mainly rest upon the before mentioned symptoms, *Voisin* again refers to its intimate relation with menstruation. He rejects an exploring puncturing, but *Braun* considers this an important means of diagnosis from ovarian tumors. This author considers it very difficult to distinguish between an effusion within, or without the peritoneum. The circumstances under which displacement of the os may take place have not been sufficiently ascertained to make this a diagnostic point. Equally difficult does *Voisin* consider the diagnosis between this and abcess. *Voisin* also gives a few points of distinction between hæmatocele and ovarian cysts, extra-uterine gestations, uterine fibroids, retroversion and retroflexion of the uterus, varices of the lig. lata, thrombus, accumulation of fæces in the rectum. *Braun* mentions that in hæmatocele he re is no appearance of any secretion of milk, which might assist in distinguishing from gravidity.

The pathological condition in hæmatocele retro-uterina is generally as follows: The quantity of the effused blood averages from 6 to 40 ounces. Bladder, rectum and vagina are involved, their walls frequently perforated, in other cases only thinned; these fistulous openings are generally obstructed by clots, the uterus by adhesions is pulled aside or entirely turned upon its axis. The uterus is frequently very voluminous, its walls softened, or infiltrated with blood.

The *prognosis* in cases of non-encysted hæmorrhages is very unfavorable. In hæmatocele, no matter how it terminates, we must always expect peritonitis and internal hæmorrhage.

The *treatment* in the larger effusions consists in the observance of the most absolute rest, the application of ice to the abdomen, and sinapisms to the upper extremities. In hæmatocele the treatment is either chirurgical, that is, an opening either by puncturation or incision is indicated, or medical, emollient poultices to the abdomen autip dogisties and, where possible tonics.

Puncturation, according to *Nelaton*, is performed with a trocar through the vagina, and the opening may be enlarged by a bistoury. *Reamur* used another method. According to *Puech* statistics speak against the operation, as in 14 cases there were five deaths, divided as follows:

| | | | |
|---|---|---|---|
| Recamier | 2; | 2 | resulted in a cure. |
| Velpeau | 1; | 1 | resulted in a cure. |
| Robert | 1; | 1 | resulted fatally. |
| Monold | 2; | 2 | resulted fatally. |
| Hagaier | 1; | 1 | resulted fatally. |
| Voisin | 7; | 2 | resulted fatally. |

*Voisin* considers internal treatment for the one alone generally applicable, by which he has only 5 deaths to 20 cures. To promote re-absorption *Braun* recommeuds inunction with iodine dissolved in glycerine. Generally the internal treatment is of course the same as that in circumscribed peritonitis and internal haemorrhage.

*Voisin* closes his monograph with a relation of 36 cases and a plate of an hematocele retro uterina andits relation to the neighboring organs,

